# Crisis and Change: Fallstudie zur Untersuchung der Resilienz als Erfolgsfaktor von Partizipationsplattformen während der Coronakrise

**DOI:** 10.1365/s40702-021-00756-6

**Published:** 2021-07-01

**Authors:** Jan-Hendrik Butt, Felix Scholz, Pascal Abel

**Affiliations:** Institut für Wirtschaftsinformatik, Technische Universität Braunschweig, Braunschweig, Deutschland

**Keywords:** Partizipationsplattformen, Resilienz, Bottom-up Urbanism, Online-Communities, Coronakrise, Participatory platforms, Resilience, Bottom-up urbanism, Online communities, Corona crisis

## Abstract

Die Coronakrise trifft große Teile der Weltwirtschaft hart und reduziert das gesellschaftliche Leben stark. Durch die stark einschränkenden Maßnahmen zur Minimierung der sozialen Kontakte wäre auch ein Rückgang des Interesses an der gemeinschaftlichen Gestaltung der städtischen Umgebung zu erwarten. Jedoch ist das Gegenteil der Fall. Partizipationsplattformen für die Gestaltung des urbanen Raumes haben sich verändert, um der Krise zu begegnen und Antworten auf die aktuellen Herausforderungen zu finden. Dieser Beitrag untersucht, weshalb Partizipationsplattformen trotz oder durch die Coronakrise erfolgreicher werden und möchte erforschen, welche Ursachen es für diese Resilienz gibt. Dafür dienen 26 Partizipationsplattformen im urbanen Raum als Untersuchungsgrundlage. Alle Plattformen bieten das höchste Partizipationslevel der Selbstverwaltung an, sodass Bürger*innen selbstständig ihren Lebensraum gestalten können. Anhand von drei Fallstudien werden Ausdauerfähigkeit, Anpassungsfähigkeit und Wandlungsfähigkeit der Plattformen beschrieben. Als Ursachen konnten dabei unter anderem die enge Verbundenheit zu den Projektinitiatoren und das starke gesellschaftliche Verantwortungsgefühl sowie flache Hierarchien und eine gelebte Fehlerkultur herausgearbeitet werden.

## Abstract (en)

The corona crisis is hitting large parts of the global economy hard and severely reducing social life. The severely restrictive measures to minimize social contacts would also lead to a decrease in interest of the shared design of the urban environment. However, the opposite is true. Participation platforms for the design of urban space have changed in order to face the crisis and find answers to the current challenges. This article examines why participation platforms are becoming more successful despite or due to the corona crisis and would like to research the causes of this resilience. For this purpose, 26 participation platforms in urban space serve as a basis for research. All platforms offer the highest level of participation in self-governance, so that citizens can design their living space independently. On the basis of three case studies, the persistence, adaptability and transformability of the platforms are described. The reasons for this were, among other things, the close ties to the project initiators and the strong sense of social responsibility as well as flat hierarchies and a lived error culture.

## Partizipationsplattformen des urbanen Lebensraumes und die Coronakrise

Bürger*innen möchten den urbanen Raum, in dem sie leben, zunehmend selbst gestalten. Sie nähen Pullover für Straßenlaternen, pflanzen Blumen in ungenutzten urbanen Räumen (Fabian und Samson 2015) oder gestalten öffentliche Gegenstände und Plätze neu. Sichtbar ist der Trend des „Do-it-yourself-Urbanismus“ (Iveson 2013) oder bottom-up Urbanism in Europa und Nordamerika. In diesen Regionen war seit der modernen Ära die Gestaltung der Stadt erst allein von der Politik geprägt und hat sich im Laufe der Jahrzehnte durch mächtige privatwirtschaftliche Interessen verändert (Arefi und Kickert 2019). Das Verlangen nach Partizipation der Bürger*innen wächst und mit ihr entspringen neue Formen wie Partizipation verwirklicht wird.

Ein weiterer Trend sind Online-Communities, in denen sich zunehmend mehr Menschen organisieren. Diese sind seit ihrer Entstehung vor über 20 Jahren mittlerweile die beliebtesten Dienste des Internets (Malinen 2015). Die wachsende Beliebtheit von Online-Communities zeigt sich auch durch Partizipationsplattformen für die Gestaltung des urbanen Raums. Diese ermöglichen es Bürger*innen, sich in einem virtuellen Netzwerk zu organisieren. Das Ziel solcher Partizipationsplattformen ist das Ermöglichen von verschiedenen Ebenen des Engagements für Bürger*innen und unterschiedlicher Intensität der Beteiligung (Falco und Kleinhans 2018). Ein Beispiel ist die Partizipationsplattform „Raumpioniere^1^“. Auf dieser können die Bürger*innen selbst Projekte initiieren, ihre Ideen durch Crowdfunding realisieren, Ideen sammeln und Mitstreiter*innen gewinnen können. Partizipationsplattformen ermöglichen ein digitales Zusammenfinden von Menschen, was gleichzeitig zu einem wachsenden Online-Netzwerk führt. Als Ergänzung von Offline-Communities fördern sie die Interaktion und erhöhen die Möglichkeiten zur Beteiligung (Gil de Zúñiga und Valenzuela 2011).

Ende des Jahres 2019 und Anfang des Jahres 2020 beginnt auf der gesamten Welt die Coronapandemie. Als Folge dessen entsteht eine weltweite Wirtschaftskrise, die insbesondere die Gastronomie und Veranstaltungsbranche trifft. Hingegen erzielen beispielsweise Internetplattformen wie Amazon ein enormes Wachstum.

Durch die starken Einschränkungen zur Minimierung der Ausbreitung des Coronavirus wäre ein Rückgang des Interesses für die gemeinschaftliche Gestaltung des urbanen Raumes auf Partizipationsplattformen zu erwarten gewesen, was sich in der Nutzeraktivität auf diesen widerspiegeln sollte. Jedoch zeigt sich am Beispiel der amerikanischen Partizipationsplattform „Ioby^2^“ (Akronym für „in our backyards“), dass genau das Gegenteil eingetreten ist. In dem Zeitraum von Januar bis November 2020 haben sich die eingeworbenen Mittel im Vergleich zum Vorjahrszeitraum mehr als verdreifacht (ca. 4,5 Mio. US $). Dabei sind ebenfalls die erfolgreich gestarteten Projekte im gleichen Zeitraum gegenüber des Vorjahreszeitraums leicht gestiegen (Ioby 2020). Es scheint, als wäre die Plattform resilient gegenüber den Auswirkungen der Coronakrise. Resilienz ist überwiegend als psychologische Widerstandsfähigkeit z. B. gegenüber Schicksalsschlägen wie dem Tod eines nahen Angehörigen bekannt. Doch auch im Zusammenhang von Systemen wird der Begriff der Resilienz genutzt z. B. als ein kontinuierliches Verändern und Anpassen, um innerhalb eines funktionierenden Stabilitätsbereiches zu bleiben (Folke et al. 2010).

Diese Arbeit fokussiert die folgende Forschungsfrage: Wie trägt die Resilienz von Partizipationsplattformen zu einem Erfolg während der Corona-Pandemie bei und worin liegen die Ursachen der Resilienz?

In dieser Studie wurden drei Plattformen betrachtet, um Merkmale von Resilienz anhand von Kategorien aus der Literatur zu untersuchen. Dabei konnte gezeigt werden, dass es sich bei den Plattformen um resiliente Systeme handelt und es wurden die Ursachen dafür aufgezeigt. Damit wollen wir den Blickwinkel der Resilienz für Praktiker*innen verdeutlichen und anhand der Ursachen und Beispiele Inspiration für die Gestaltung der eigenen Plattformen geben.

## Resilienz von Partizipationsplattformen und das Forschungsvorgehen

Eine schwerwiegende Krise, wie sie die Corona-Pandemie darstellt, führt zu Veränderungen, die Systeme, wie z. B. Partizipationsplattformen, betreffen. Sie können dazu führen, dass bisherige Angebote und Geschäftsmodelle ausgehebelt werden. Wenn die Reaktionen und entsprechenden Anpassungen dazu führen, dass die Plattformen wieder erfolgreich sind und weiterhin bestehen können, sprechen wir von Resilienz. Nach Folke et al. (2010) setzt sich Resilienz aus drei Aspekten zusammen: Ausdauerfähigkeit (engl. persistence), Anpassungsfähigkeit (engl. adaptability) und Wandlungsfähigkeit (engl. transformability). Unter Ausdauerfähigkeit ist die Fähigkeit zu verstehen, innerhalb eines Stabilitätsbereichs trotz störender Faktoren zu bleiben. Die Anpassungsfähigkeit ist die Fähigkeit, Antworten auf sich verändernde externe Einflüsse und interne Prozesse zu finden, wobei diese Veränderungen innerhalb des derzeitigen Stabilitätsbereichs und Entwicklungspfads stattfinden (Folke 2010). Die Wandlungsfähigkeit geht darüber hinaus und liegt vor, wenn neue Entwicklungspfade beschritten werden, wodurch neue Stabilitätsbereiche geschaffen sowie bestehende Schwellenwerte überschritten werden. Ein Beispiel, welches diese Fähigkeit verdeutlicht, bietet Amazon. Neben dem Marketplace ist Amazon mit der Sparte „Web Services“ einer der führenden Dienstleister für Cloud-Computing. Dies zeigt die Wandlungsfähigkeit des Unternehmens, das sich von einem Onlineversandhändler zu einem der führenden Technologieunternehmen der Welt entwickelt hat.

Die Coronakrise ist die Ursache für tiefgreifende Veränderungen, die unter anderem die Wirtschaft und das gesellschaftliche Zusammenleben stark beeinflussen und sich damit direkt auf das System einer Partizipationsplattform auswirken. Die Auswirkungen sollen anhand des Erfolges betrachtet werden. Hier messen wir den Erfolg anhand der selbstgesetzten Ziele sowie positiver Entwicklungen, die sich während der Krise ergeben haben.

Für die Untersuchung der Resilienz als Erfolgsfaktor von Partizipationsplattformen wurde ein empirischer, qualitativer Forschungsansatz gewählt. Aufgrund der Aktualität der durch die Coronakrise entstandenen Wirtschaftskrise eignet sich besonders der Einsatz von Fallstudien. Konkret werden drei Partizipationsplattformen ausgewählt, die auf ihrer Webseite Anpassungen in Bezug auf die Coronakrise vorgenommen haben. Um eine fundierte Datenbasis zu erheben, werden halb-strukturierte Interviews geführt, die darauf abzielen Resilienz zu ermitteln, Erfolg über den Zeitraum der Coronakrise festzustellen und herauszufinden, ob der Erfolg aus der Resilienz resultiert. Zur Auswertung der Daten wird die Methode der qualitativen Inhaltsanalyse nach Mayring (2010) genutzt. Zuletzt werden die Ergebnisse der Datenanalyse interpretiert und zur Beantwortung der Forschungsfrage herangezogen.

Als Grundlage dient eine Datenbank mit 25 Partizipationsplattformen. Die Datenbank wurde erstmalig in einer Studie von Abel et al. (2020) genutzt und für diese Forschung übernommen. Aus dieser Datenbank heraus werden die Internetauftritte der Plattformen hinsichtlich ihrer Anpassungsfähigkeit gemessen und bewertet. Dafür wurde die PRISMA-Methode nach Moher et al. (2009) gewählt.

Hierbei wird die gesamte Menge an Webseiten strukturiert verkleinert. In Folge dieser Methode werden im ersten Schritt alle inaktiven Webseiten entfernt. Anschließend werden nur die Webseiten betrachtet, die auf die Coronakrise in einem Banner, Blogpost, Kurzmeldung hinweisen oder durch andere Finanzierungen darauf reagieren (verbleibend neun). Die Inhalte der verbleibenden Webseiten werden von zwei Forschern anhand einer Skala (eins bis fünf) klassifiziert, um die Relevanz für Interviews herauszufiltern. Dabei führt die Erwähnung von der Coronakrise mindestens zu einem Rating von eins. Wenn über die Erwähnung hinaus Tipps, Verhaltensrichtlinien oder Hervorhebungen für Corona-Projekte implementiert wurden, wird jeweils ein höheres Ranking erzielt. Das bestmögliche Ranking (fünf) wurde erzielt, wenn die Plattformbetreiber ihr Geschäftsmodell aufgrund von Corona angepasst haben. Eine solche Anpassung des Geschäftsmodells lag bei der Plattform Raumpioniere vor, dabei wurde auf die üblichen Provisionen verzichtet und die gesammelten Mittel vollständig den Projektinitiatoren überlassen. Der Mittelwert aus den Klassifizierungen der beiden Forscher bildet die finale Klasse einer Partizipationsplattform, anhand derer sie sich mit den anderen neun Plattformen vergleichen lässt.

Die dadurch entstandene Ordnung wird genutzt, um auszuwählen, welche Plattform für eine Fallstudie geeignet ist. Die neun Plattformen gut-für-Nürnberg, Hannovermachen, Ioby, Patronicity, Raumpioniere, Rabryka, Sandkasten, Spacehive, Wechange, zeichnen sich durch ein hohes Maß an Anpassungsmaßnahmen auf ihrer Webseite aus. Für ein Interview im Rahmen der Fallstudien haben sich drei Plattformen bereiterklärt. Zur detaillierteren Datenerhebung sollen in einem darauffolgenden Schritt halb-strukturierte Online-Interviews mit den Partizipationsplattformen Sandkasten, Raumpioniere und Rabryka geführt werden, die somit als Gegenstand der Fallstudien zu untersuchen sind.

Das in Braunschweig agierende Projekt Sandkasten wurde 2015 an der Technischen Universität Braunschweig ins Leben gerufen^3^. Schon im Jahr 2003 entstand aus einer Dresdner Jugendbewegung in Görlitz das Projekt Rabryka^4^. Die jüngste dieser Partizipationsplattformen sind die Wiener Raumpioniere, die 2017 gegründet wurden^5^.

Die Inhaltsanalyse der Interviews erfolgt nach Mayring (2010) qualitativ orientiert. Die Kategorien werden anhand eines deduktiven Prozesses gebildet, was bedeutet, dass die Daten den vorab definierten Kategorien „Erfolg“, „Resilienz“ und „Ursache der Resilienz“ zugeordnet werden. Die Kategorien leiten sich aus der Fragestellung sowie der Literatur zu Erfolg und Resilienz ab und finden Berücksichtigung im Interviewleitfaden bei der Datenerhebung.

## Nachweise für Resilienz und die Ursachen

Die Partizipationsplattformen Sandkasten, Raumpioniere und Rabryka wurden interviewt, um herauszufinden wie Veränderungen zu Anpassungen führen.

Der nachfolgenden Tab. 1 sind die Kategorien (K01 bis K06) aufgelistet, die als Nachweise für Resilienz untersucht wurden und die Ursachen (U07 bis U09) zur Resilienz dargestellt. Durch Kreuze wurde markiert, wenn die untersuchten Aspekte nachgewiesen wurden. So erfüllt beispielsweise das Erreichen der gesetzten Ziele die Kategorie K01: Erfolg.

**Tab. 1:** Vergleich der Plattformen

Kategorie	Sandkasten	Raumpioniere	Rabryka
K01: Erfolg	–	–	X
K02: Erfolg (Nutzerverhalten)	X	X	–
K03: Ausdauerfähigkeit	X	X	–
K04: Anpassungsfähigkeit Projekte	X	X	X
K05: Anpassungsfähigkeit Plattform	X	X	X
K06: Wandlungsfähigkeit	X	X	–
U01: Ursachen für Ausdauerfähigkeit	X	X	X
U02: Ursachen für Anpassungsfähigkeit	–	X	X
U03: Ursachen für Wandlungsfähigkeit	X	–	X

Der gewählte Ansatz zur Feststellung von Erfolg fundiert auf der Betrachtung zweier Merkmale. Zuerst, ob die von den Plattformen an sich selbst gesetzten Ziele erreicht wurden. Die zweite Ausprägung von Erfolg beruht auf den positiven Entwicklungen, die innerhalb von Projekten, welche während der Coronakrise gestartet wurden, sichtbar geworden sind. Dabei wurde beispielsweise in Betracht gezogen, ob eine außerordentliche Aufmerksamkeit in der Presse aufgetreten ist oder ein starkes Wachstum anhand von Nutzerzahlen festgestellt werden konnte.

### K01: Die Ziele der Plattform konnten erreicht werden

Die Plattform Rabryka konnte über die letzten Jahre ein starkes Wachstum an Projekten, Nutzern und Fördermittelgeber verzeichnen. Die Coronakrise verlangsamte dieses Wachstum. Dennoch wurde das Ziel der Plattform „Fördermittel bekommen und Bedürfnisse der Görlitzer befriedigen“ erreicht. Rabryka meint dazu: „Die Pandemiezeit hat nicht geschadet, wir haben die aktiven Leute nicht verloren, keine Förderungen eingebüßt und allgemein keinen Nachteil bekommen.“ Die Ziele der Plattformen Sandkasten und Raumpioniere sowie die Erreichung dieser konnte in den Interviews nicht erfasst werden, daher konnte die Erfüllung dieser Kategorie den Plattformen nicht nachgewiesen werden.

### K02: Kurzfristige außerordentliche Aufmerksamkeit für soziale Projekte

Die Plattformen Sandkasten und Raumpioniere zeigen beide Veränderungen im Nutzerverhalten. Soziale Projekte, die als Reaktion auf Herausforderungen durch die Coronakrise entstanden sind, haben eine extrem hohe Aufmerksamkeit erhalten. Das Homestage Festival, wo „verschiedenste lokale Künstler*innen [Wiens] ihre Performances live aus ihrem Zuhause ins Zuhause des Publikums streamen“^6^, konnte nach Angabe der Raumpioniere im Vergleich zu normalen Projekten circa das 300-fache an Aufrufen erzielen. Gleichzeitig berichtet der Sandkasten aus Braunschweig, dass die gewonnene Aufmerksamkeit nur bestimmten Projekten zuteilwurde. Das „Grundrauschen“ der Besuche der Plattform sei im Vergleich zu den vorherigen Jahren niedriger ausgefallen.

Nach der Betrachtung des Erfolgs der Plattformen werden im Anschluss die Merkmale von Resilienz untersucht. Diese teilen sich in Ausdauerfähigkeit, Anpassungsfähigkeit und Wandlungsfähigkeit auf und wobei wir unabhängig voneinander die Anpassungsfähigkeit der Projekte (K04) und der Plattform (K05) betrachtet haben.

### K03: Plattformbetreiber sind um Projekterfolge gewillt und tragen zum Lösen von Problemen bei

Alle interviewten Plattformen pflegen eine Verbindung zu Projektinitiatoren oder sind diese sogar selbst. Bei der Plattform Sandkasten zeigt sich dies darin, dass Langzeitprojekte, wie der Bau des Studierendenpavillons, trotz vieler Widrigkeiten dennoch erfolgreich umgesetzt wurden. Auch das Neuaufstellen des Projektteams durch Sandkasten sei bereits vorgekommen, wenn Projektinitiatoren ihr Studium beendet haben und das Projekt nicht fortführen konnten. Die Plattformbetreiber von Raumpioniere motivieren die Projektinitiatoren, wenn es notwendig ist, bis zu einem gewissen Grad und stehen ihnen beratend zur Seite.

### K04: Starke Ausrichtung der Projekte auf die Coronakrise

Die während der Coronakrise initiierten Projekte sind auf allen Plattformen auf die Herausforderungen der Pandemie angepasst oder sollen ihnen direkt entgegenwirken. Auf der Plattform Sandkasten wurden zum Beispiel das Projekt „behelfs.mundschutz.fuer.braunschweig“, bei dem ehrenamtliche Näher*innen Ende März 13.000 Masken genäht und an 160 Institutionen in Braunschweig verteilt haben oder die Initiative „Hey, Alter! Alte Rechner für junge Leute“, welche ungenutzte Desktop PCs und Laptops von Firmen und Privatpersonen einsammelt und Schüler*innen aus einkunftsschwachen Familien für Homeschooling überlässt, initiiert. Bei anderen Projekten wurden Sicherheitskonzepte entwickelt, um die Beschränkungen einzuhalten oder Zusammenkünfte digital abzuhalten, was eine Anpassung an die Situation darstellt.

### K05: Neue Angebote, die auf die Auswirkungen Coronakrise zugeschnitten sind, Anpassung der internen Struktur und Ausrichtung (bestehendes Feld ausbauen)

Neben der Anpassung der Projekte auf die Coronakrise haben auch die Plattformen ihre Angebote angepasst. Die Plattform Raumpioniere verzichtet während der Pandemie auf Einnahmen durch Provisionen. Rabryka hat die Bedeutung von Social Media verstärkt wahrgenommen und entschieden, sich hinsichtlich der Form und Frequenz des Social Media Outputs neu zu positionieren. Die Plattform Sandkasten hat neben einem neuen Modul „Helfende Hände“, wo Nutzer ohne eigenes Projekt ihre Fähigkeiten anbieten können, auch intern weitere neue Strukturen gebildet.

### K06: Öffnung der Plattform für neue Inhalte, Transformation der internen Struktur und Ausrichtung (neues Feld erschließen)

Die Plattformen Sandkasten und Raumpioniere bemerkten einen spürbaren Wandel zu sozialeren Projektthemen. „Das Urbane war zwar noch ein Anhängsel bei einigen Projekten, aber das Soziale stand bei den meisten im Vordergrund“, berichten die Raumpioniere. Von einer Veränderung vom ursprünglichen Ziel, als Plattform zur Campusgestaltung hin zur Plattform für soziale Projekte und Campusgestaltung, berichtete auch die universitätsnahe Plattform Sandkasten. Dieser Wandel der Projektthemen beschreibt eine Öffnung der Plattformen für neue Zielgruppen, ohne den alten Stabilitätsbereich zu verlassen. Es handelt sich somit um eine Transformation im Sinne der Resilienz nach Folke et al. (2010). Gleichzeitig könnte sich dieser Wandel vor der Coronakrise abgezeichnet haben oder sogar als langfristige Strategie festgelegt worden sein, doch gleicht die Rolle der Pandemie der eines Katalysators, der diesen Wandel beschleunigt.

Nachdem festgestellt wurde, dass die drei Plattformen resiliente Systeme sind, wurden die Ursachen (U01 bis U03) für die Resilienz untersucht.

### U01 Ausdauerfähigkeit: Enge Verbundenheit zu den Projektinitiatoren und Verantwortungsgefühl

Die Verantwortung des Projekterfolges obliegt den Initiatoren, dennoch existiert eine enge Zusammenarbeit zwischen ihnen und den Plattformbetreibern. Die Plattform Raumpioniere beschreibt die Zusammenarbeit wie folgt: „Wir sind immer mit dabei, beim Launch, beim Start oder beim Abschluss des Projektes. Insofern gibt es keinen Evaluierungskatalog. Es gibt aber den stetigen Kontakt und somit haben wir immer ein Auge auf die Projekte“. Sie betreuen die Projektinitiatoren, indem sie eine beratende Funktion einnehmen, die Verantwortung liegt dennoch beim Projektinitiator. Das Team um Rabryka initiiert selbst Projekte, für die sie die volle Verantwortung tragen. Durch die Nähe zu den Projekten, ist die Wichtigkeit von Ausdauerfähigkeit bekannt und wird selbst erfahren. Das Rabryka Team nimmt selbst die Nutzerrolle ein und trainiert so die eigene Ausdauerfähigkeit und kann authentischer die Situationen, in denen Durchhalten gefragt ist, erkennen und vermitteln. Durch enge Verbundenheit zu den Initiatoren wird eine Beziehung zwischen Rabryka und den Projektteams aufgebaut, die ein erhöhtes Verantwortungsgefühl erzeugt und somit die Ausdauerfähigkeit beider Seiten steigert. Bei Sandkasten ist eine enge Verbindung von den Mitarbeitenden zu den Projektteams vorhanden und die Mitarbeitenden sind häufig selbst ehrenamtlich aktiv. Auch zeigt sich die Ausdauerfähigkeit der Projektteams dadurch, dass nach dem Abschluss des Studiums die Projekte fortgeführt und betreut werden.

### U02 Anpassungsfähigkeit: Kein ökonomischer Druck und kurze Entscheidungswege

„Wir sind anpassungsfähig, weil wir keinen ökonomischen Druck verspüren“, berichten die Raumpioniere. Das Team sei nicht auf die Einnahmen der Plattform angewiesen, weshalb es möglich ist, ein Angebot wie den Verzicht auf Provision aus Projektfinanzierungen umzusetzen. Nicht zwingend einen bestimmten Gewinn erzielen zu müssen, ermöglicht es, riskantere Entscheidungen zu treffen und damit Auswirkungen besser entgegenzuwirken. Ein solcher ökonomischer Druck kann entweder negativ zu einer Schockstarre führen oder zu positiver Schaffenskraft antreiben. Darüber hinaus heben die Raumpioniere die flachen Strukturen der Plattform hervor, die der Grund sind, weshalb Entscheidungen schnell getroffen werden können. Die Plattform Rabryka pflegt eine Grundphilosophie des „Trial-and-Error“. Projektangebote werden retrospektiv bewertet und daraus werden die nächsten Schritte abgeleitet. Wenn es von der Community angenommen wird, wird es in der Folge verbessert, vergrößert oder standardisiert. Sollte die Reaktion der Community zurückhaltend oder gar negativ ausfallen, wird das Projektangebot überdacht, verbessert oder fallen gelassen. Die Community dient als Indikator für die Bewertung von Projekten. Außerdem wird damit eine Fehlerkultur geschaffen, wobei Fehler als wichtiger Bestandteil für die Entwicklung der Plattform gesehen werden.

### U03: Wandlungsfähigkeit liegt in der DNA der Plattform

Rabryka ist aus einem Jugendbegehren und einem Flashmob im Stadtrat entstanden und umfasst mittlerweile 15 Angestellte, erhält Förderungen von der Stadt Görlitz, dem Land Sachsen und der Europäischen Union und hat eine professionelle Struktur. Diese Entwicklung spiegelt die Transformationsfähigkeit der Plattform wider. Bei Sandkasten liegt Transformationsfähigkeit in Mitarbeitern und Benutzern begründet. Sie haben gemeinsam während der Coronakrise aktiv institutionelle und ehrenamtliche Kapazitäten zum Wohle ihrer Community einsetzen und andere Aufgaben ruhen lassen können. Das Sandkastenteam fördert dies durch Offenheit und Unvoreingenommenheit gegenüber eingebrachten Ideen und Impulsen.

## Übertragbarkeit in die Praxis und die Möglichkeiten zur weiteren Forschung

Die Aspekte Ausdauerfähigkeit, Anpassungsfähigkeit und Wandlungsfähigkeit haben dazu beigetragen, dass die Plattformen trotz äußerer Einschränkungen durch die Coronakrise Erfolge erzielen konnten. Die Partizipationsplattformen Sandkasten, Raumpioniere und Rabryka erfüllen somit die Definition eines resilienten Systems. Dies zeigt sich beispielsweise in der engen Verbindung zu den Projektinitiatoren und einem hohen Empfinden von Verantwortlichkeit (Ausdauerfähigkeit), besonderen Angeboten, die auf Projekte mit Bezug zu Corona zugeschnitten werden (Anpassungsfähigkeit der Plattform), sowie die Öffnung der Plattform für gesamtgesellschaftliche Projekte (Wandlungsfähigkeit) und die Ausrichtung der Projektinhalte auf die Problematiken der Coronakrise (Anpassungsfähigkeit der Inhalte).

Die Ursachen der Resilienz finden sich in verschiedenen Aspekten. Die Ausdauerfähigkeit resultiert aus einer engen Verbundenheit zu den Projektinitiatoren und einem starken Verantwortungsgefühl für die Gesellschaft und den Projekterfolg. Eine Ursache für Anpassungsfähigkeit findet sich in der finanziellen Aufstellung, die möglichst keinen Druck auf die Entscheidungen ausüben sollte. Außerdem tragen kurze Entscheidungswege und eine Kultur der Fehlertoleranz zur Anpassungsfähigkeit bei. Die Ursache für Wandlungsfähigkeit liegt in der DNA der Plattform. Sollte sie sich häufigen Veränderungen und Transformationen unterziehen und ihre Position sowie ihre Motivation häufig hinterfragen, ist sie gewillter, sich einer Transformation zu unterziehen.

Die Ergebnisse dieser Arbeit könnten dazu genutzt werden, um kurzfristig zu resilienten Maßnahmen zu inspirieren oder die Resilienz langfristig zu verstärken. Auch das Vorgehen aus dieser Arbeit mit dem Fokus Krisen zu überwinden, kann auf zukünftige Arbeiten in Kontexten abseits von Partizipationsplattformen genutzt werden.

Bei Betrachtung der Ergebnisse ist sowohl zu berücksichtigen, dass ein Interviewer-Bias vorliegt, als auch dass die Ergebnisse der subjektiven Meinung der interviewten Experten entstammen. Eine weitere Limitation besteht darin, dass lediglich drei Partizipationsplattformen untersucht wurden. Die Ergebnisse sind deshalb nicht repräsentativ. Sie können jedoch als Grundlage für die Herleitung von Hypothesen bei umfangreicheren Studien dienen. Das Auswerten des Verhaltens in einer Krise, wenn die Krise noch nicht durchgestanden ist, ist ein weiterer Punkt der Unsicherheit. Aktuell können die Plattformen als resilient bezeichnet werden, jedoch könnte ein längeres Anhalten der Krise neue Herausforderungen bereithalten, die nicht überwunden werden können.

Zukünftige Arbeiten sollten versuchen, ein Verfahren zur Messung der Resilienz für Partizipationsplattformen zu verbessern und zu objektivieren. Diese Verfahren könnten von den Plattformen selbst genutzt werden, um beispielsweise Krisen widerstandsfähiger zu begegnen. Durch eine Selbstbewertung könnten Stärken und Schwächen aufgedeckt und geeignete Maßnahmen zur Erhöhung der Resilienz entwickelt werden.

## Footnotes

1 https://raumpioniere.at/.

2 https://ioby.org/.

3 https://www.sandkasten.tu-braunschweig.de/sandkasten.

4 https://www.rabryka.eu/Verein.html.

5 https://raumpioniere.at.

6 https://raumpioniere.at/de/homestagefestivals.
